# *Insilico* targeting of virus entry facilitator NRP1 to block SARS-CoV2 entry

**DOI:** 10.1371/journal.pone.0310855

**Published:** 2025-02-05

**Authors:** Nousheen Bibi, Maleeha Shah, Shahzad Khan, Muhammad Shahzad Chohan, Mohammad Amjad Kamal

**Affiliations:** 1 Departments of Bioinformatics, Shaheed Benazir Bhutto Women University Peshawar, Peshawar, Pakistan; 2 Department of Biomedical Sciences, College of Clinical Pharmacy, King Faisal University, Al Hofuf, Al-Ahsa Saudi Arabia; 3 Institutes for Systems Genetics, Frontiers Science Center for Disease-related Molecular Network, West China Hospital, Sichuan University, Chengdu, China; 4 King Fahd Medical Research Center, King Abdulaziz University, Jeddah, Saudi Arabia; 5 Faculty of Allied Health Sciences, Department of Pharmacy, Daffodil International University, Dhaka, Bangladesh; 6 Enzymoics, Novel Global Community Educational Foundation, Hebersham, NSW, Australia; D Y Patil Deemed To Be University, INDIA

## Abstract

The entry and infectivity of a virus are determined by its interaction with the host. SARS-CoV-2, the virus responsible for COVID-19, utilizes the spike (S) protein to attach to and enter host cells. Recent studies have identified neuropilin-1 (NRP1) as a crucial facilitator for the entry of SARS-CoV-2. The binding of the spike protein to the b1 domain of NRP1 has been shown to enhance viral infection twofold. Consequently, targeting NRP1 to disrupt this interaction represents a promising strategy to mitigate viral infection. In this study, a small molecule library of approximately 10,000 compounds was screened to identify those that could inhibit the interaction between NRP1 and the spike protein by targeting the b1 domain of NRP1. The crystallographic structure of the b1 domain of human NRP1 (PDB entry: 7JJC) was used for this purpose. Following virtual screening, docking studies, and evaluation of binding affinity and ADMET properties, 10 compounds were shortlisted. The top two candidates, AZD3839 and LY2090314, were selected for molecular dynamics simulation studies over 100 ns to assess binding stability. MM/GBSA calculations indicated that both AZD3839 and LY2090314 exhibited strong and stable binding to the b1 domain of NRP1. Computational modeling of the interaction between the b1 domain of NRP1 and the receptor-binding domain of the spike protein suggested that AZD3839 and LY2090314 could effectively hinder the NRP1-spike interaction. Therefore, these compounds may serve as potential drug candidates to reduce SARS-CoV-2 infectivity.

## Introduction

The global healthcare systems, economies, and social fabric have faced profound challenges due to the emergence of the 2019 coronavirus disease (COVID-19), caused by the novel severe acute respiratory syndrome coronavirus 2 (SARS-CoV-2) [1,2] Belonging to the family of Coronaviridae, the coronavirus is known to induce respiratory, digestive, and neurological illnesses in both humans and numerous animal species [3–5]. COVID-19 primarily affects the respiratory system and is typically spread from person to person through respiratory droplets and direct contact with individuals infected with SARS-CoV-2 or contaminated surfaces [1,6–8] A similar virus, SARS-CoV, caused a significantly smaller outbreak in 2003, potentially because it primarily infected the lower respiratory system. In contrast, SARS-CoV-2 spreads quickly through active viral shedding in the pharynx [9]. Despite these variances, both viruses utilize the identical cellular receptor, known as a angiotensin-converting enzyme 2 (ACE2), for entry [8,10–12]

Research focusing on elucidating the function of angiotensin-converting enzyme 2 (ACE2) as a key entry receptor for SARS-CoV-2 has been pivotal during this pandemic. Presently, there is emerging evidence indicating a diminished presence of ACE2 within pulmonary tissues, contrasted with elevated levels observed in the kidneys and intestines. Consequently, there is speculation surrounding additional mechanisms governing the interaction between the virus and the host cell. Notably, co-receptors and binding factors, such as neuropilins, have been identified, suggesting a more complex process at play [13–16]. The spike protein of SARS-CoV-2 has been suggested to harbor a furin cleavage site, which could potentially produce a C-terminus (CendR). Molecular modeling predictions indicate that this CendR might have the ability to bind to the b1 domain within neuropilin-1 (NRP1) [17,18]. This might represent an additional aspect of the COVID-19 infection process, indicating that certain factors within the population lead to a specific attraction of SARS-CoV-2 due to the presence of ACE2 and NRP1 proteins on the cell membrane, which interact with the Spike protein. Consequently, neuropilin-1 has assumed a significant role in COVID-19, prompting further investigation to understand its impact on the infectious mechanism of this disease [19,20].

NRP1 functions as a transmembrane receptor, primarily serving as a co-receptor for various ligands. Due to its absence of a cytosolic protein kinase domain, it triggers a diverse array of effects, including but not limited to cell proliferation, angiogenesis, and regulation of axon guidance [21–23]. NRP1, part of a family of signaling and catalytic proteins, has recently been demonstrated to act as an entry factor, enhancing the infectivity of SARS Coronavirus 2 (SARS-CoV-2) in vitro [13,18]. Neuropilins are exclusive to vertebrates, and thus far, homologs of NRP have been partially or fully identified in zebrafish, frogs, chicks, mice, rats, and humans, exhibiting diverse expression patterns across different species [24]. NRP1 exists in two isoforms: one is a truncated, secreted form, while the other is a transmembrane form that engages with SARS-CoV-2 [13]. After being infected, the SARS-CoV-2 Spike (S) protein undergoes cleavage by the host cell protease furin, forming S1 and S2 polypeptides. This process exposes the CendR motif within the S1 polypeptide [18]. This motif is termed the "C-end terminal rule," which necessitates the inclusion of a cationic amino acid, typically arginine, at the carboxyl terminus of the ligand, leading to an RXXR configuration). The binding pocket for CendR is located within the b1 domain of NRP1 [25]. Recently, Daly et al. demonstrated that the CendR motif within the S1 protein of SARS-CoV-2 interacts with NRP1, thereby enhancing the virus’s infectivity. Furthermore, they found that a monoclonal antibody targeting the extracellular CendR binding site within the b1 domain of NRP1 effectively decreases SARS-CoV-2 infectivity [18]. Examination of NRP1 RNA expression in cells extracted from bronchioalveolar lavage of individuals with severe COVID-19 revealed heightened levels in cells infected with SARS-CoV-2, as opposed to uninfected cells [13,26].

In this study, we screened a small molecule library consisting of approximately 10,000 compounds against the b1 domain of NRP1. The aim was to identify potential inhibitors disrupting the interaction between NRP1 and the spike protein. Following screening, compounds that showed promising inhibitory activity were subjected to ADME (absorption, distribution, metabolism, and excretion) and toxicity assessments. The most potent inhibitors were further investigated through molecular dynamics simulations to gain insights into the structural mechanisms underlying their interaction with NRP1. A hypothetical model depicting the binding of inhibitors to NRP1, validated by their disruption of the NRP1-spike protein interaction, suggests a promising strategy for mitigating infection.

## Material and methods

### Selection of NRP1 as drug target

An extensive examination of the literature indicates that Neuropilin-1 (NRP1) functions as a facilitator for virus entry [13,18]. The co-expression of NRP1 alongside ACE2 and TMPRSS2 may enhance the susceptibility to SARS-CoV-2 infection [13]. Tissue specificity is very important in viral infection as it depends upon the expression of entry receptors. NRP1 is present in various cell types throughout the body, with predominant expression observed in cells located in the lung, nose, and brain. Specifically, it is found in the respiratory and olfactory epithelium, as well as within the central nervous system (CNS) [13] Daly et al. reported the binding of furin-cleaved S1 fragment of the spike protein with b1 domain of NRP1 and this interaction boost the viral infection [18].

### Preparation of receptor protein

The high-resolution crystallographic structure of the b1 domain of Human Neuropilin-1 (NRP1) was retrieved from the Protein Data Bank (PDB: 7JJC) (https://www.rcsb.org). Using the UCSF Chimera visualization tool [27] the receptor protein was refined through the removal of water molecules, alongside the addition of hydrogen atoms and charges. The energy minimization and protonation were done with default parameters using the AMBER ff14SB force field [28]. The binding region was defined encompassing Tyr297, Trp301, Thr316, Ser346, Thr349, Lys351 and Tyr353 residues of NRP1. These residues are part of the b1 domain of NRP1, which plays a crucial role in the protein’s function and interaction with other molecules. Previous studies and structural analyses have indicated that these residues are involved in the interaction with the SARS-CoV-2 spike protein, as a CendR binding motif thus making them key targets for inhibiting viral entry [18].

### Screening libraries

The drug libraries used for virtual screening were sourced from SelleckChem (https://www.selleckchem.com/), including an FDA-approved library of 3035 compounds, a bioactive compound library of 5309 compounds, a natural organic compound library of 1106 compounds, and a COVID-19-related antiviral compound library of 700 compounds. These libraries were combined into a single collection for further processing using the PyRx virtual screening tool (https://pyrx.sourceforge.io/). Initially, all compounds underwent energy minimization before being prepared for molecular docking studies.

### Virtual screening

Virtual screening was conducted against the CendR binding pocket of NRP1. Molecular docking experiments were performed with the receptor kept rigid, allowing flexibility for all ligand atoms with the maximum number of rotatable bonds. High-throughput screening against the NRP1 binding site of the SARS-CoV-2 spike protein was performed using the PyRx Virtual Screening Tool (https://pyrx.sourceforge.io/). A grid box was positioned around the binding residues of the receptor molecule, with the x-dimension set to 50 points, and the y and z dimensions set to 38 and 46 points, respectively. The grid box was centered at coordinates (x: 14.194, y: 2.917, z: 11.444) with a spacing of 0.375 Å. Following grid box specification, molecular docking was performed with 100 docking runs for each ligand.

### Selection and analysis of compounds

To identify the most promising compounds from the four libraries, we evaluated the binding affinity of each compound with NRP1, considering the standard deviation for each. Molecular complexes of the top 20 compounds with NRP1 were generated using the PyMOL molecular visualization system (https://pymol.org/). These complexes were then analyzed using the BIOVIA Discovery Studio Visualizer (https://discover.3ds.com/discovery-studio-visualizer) and the UCSF Chimera program (https://www.cgl.ucsf.edu/chimera/).

### Compounds property calculation and QSAR modeling

SwissADME (http://www.swissadme.ch/index.php) a web tool and Molinspiration software (https://www.molinspiration.com) were used to calculate the physiochemical properties such as molecular weight in grams/mol, and topological polar surface area (TPSA, Å^2^), drug likeliness considering Lipinski’s ROF and oral bioavailability, pharmacokinetics (blood-brain barrier permeability, gastrointestinal absorption, and skin permeation (log K_p_), of the selected compounds were calculated. While the water solubility (LogS) and lipophilicity (LogP) were estimated using the E-Dragon 1.0 software (http://www.vcclab.org/lab/edragon/).

### Molecular dynamic simulation

Molecular Dynamics (MD) simulations were conducted in the GROMACS package version 5.0.5 [29] to assess the stability and structural behavior of the selected compounds in a complex with NRP1. The PRODRG server [30] was utilized to generate topologies for the identified high-binding affinity compounds. The GROMOS96 43a1 forcefield [31] was employed to optimize the parameters of the receptor-ligand interactions. Periodic boundary conditions were employed for non-bonded interactions, while the particle mesh Ewald method [32] was utilized to handle strong electrostatic interactions. The system was neutralized using Na+Cl salt. Energy minimization of the prepared systems was performed using the steepest descent integrator for 5000 steps.

The NVT (number of atoms, volume, temperature) ensemble was employed to stabilize the system temperature at a constant value of 300K. Pressure equilibration was carried out under the NPT (constant pressure, constant temperature) ensemble, maintaining a constant number of particles, pressure (1 bar) [33], and temperature. Simulations were conducted for a duration of 100ns. Post-simulation analysis of the resulting systems for stability and fluctuations was conducted using PyMol (http://www.pymol.org) and GROMACS tools.

### MM-PBSA calculations

Binding free energies serve as predictive measures for the affinities between receptor-ligand complexes. To assess the binding free energy of selected drugs with NRP1 molecules, calculations were performed using the Molecular Mechanics Poisson-Boltzmann Surface Area (MM-PBSA) [34,35]. A total of 100 frames from simulation trajectories were chosen to determine the binding free energy, employing the following equation

ΔGBinding=ΔGComplex–ΔGReceptor–ΔGInhibitor

Several energy components were computed: van der Waals energy, electrostatic energy, internal energy, and solvation energy. In the MM-PBSA calculations, the internal dielectric constant and external dielectric constant were set to 1.0 and 80.0 respectively, with the reciprocal grid spacing of 1.0 Å.

### Comparative docking of known NRP1 inhibitors with screening hits

The two known NRP1 inhibitors, EG00229 [36]) and EG01377 [37] were selected to dock against NRP1 to evaluate the accuracy of the docking procedure. After docking the docked complexes were superimposed with shortlisted docked complexes of AZD3839 and LY2090314. To eliminate the biases of the docking program and to crosscheck the consistency of the results we used PatchDock and FireDock (bioinfo3d.cs.tau.ac.il/PatchDock) to perform the redocking of shortlisted compounds and known NRP1 inhibitors.

### Molecular docking of apo and bound NRP1 with spike protein

The b1 domain of NRP1 in unbound state and bound state (NRP1-shortlisted compounds) were docked against the receptor binding domain (PDB:7B27) of SARS-CoV-2 spike protein using AutoDock 4.2 (https://autodock.scripps.edu/). The results were analyzed in the UCSF chimera.

## Results

### NRP1 as a drug target against SARS-CoV-2

**Fig 1:**
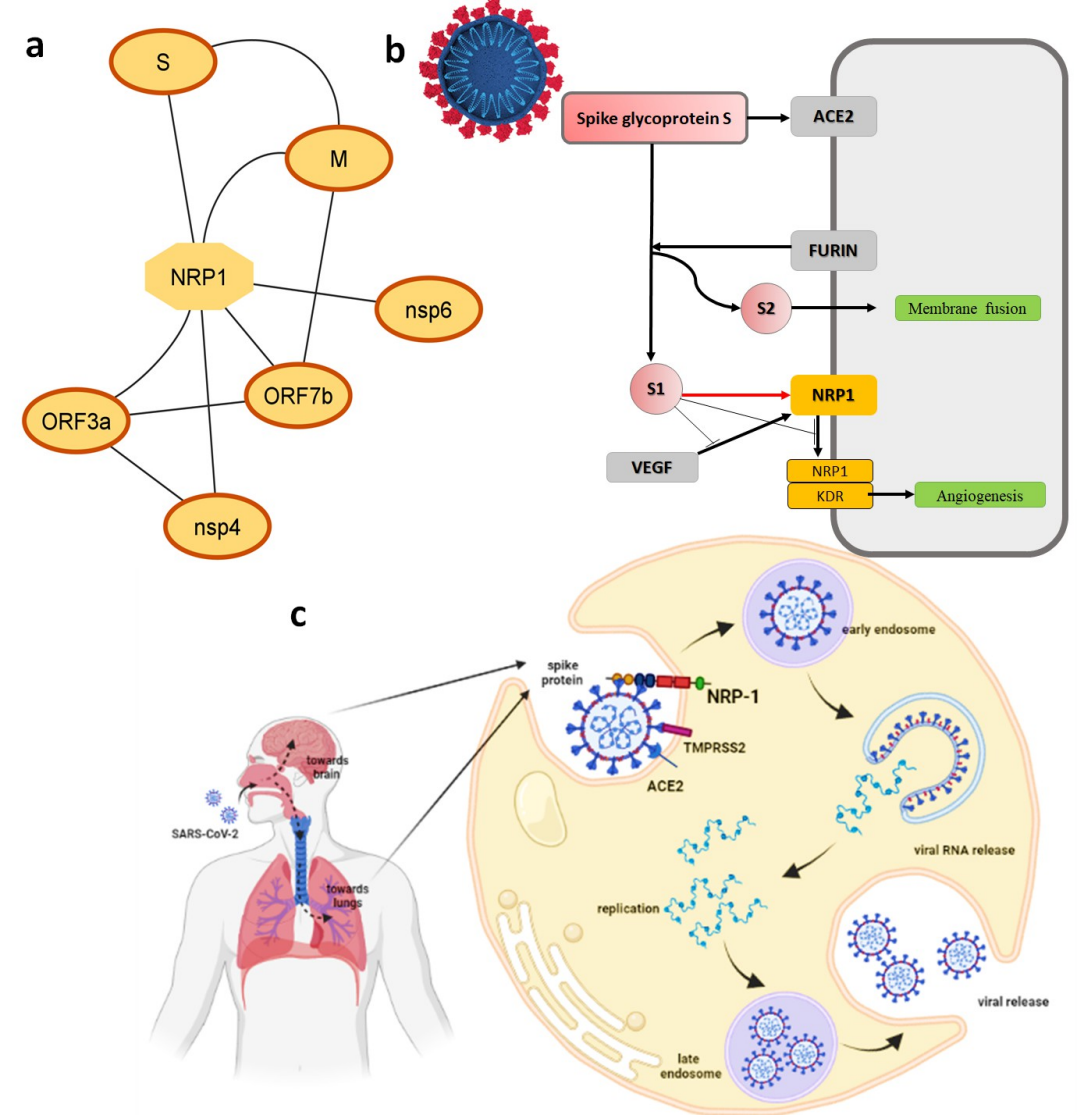
Role of NRP1 in SARS-COV-2 infection. **(a)** Interactions of NRP1 with SARS-COV-2 proteins **(b and c)** Mechanism of NRP1 as entry factor for SARS-COV-2 infection.

Several recent studies reported that NRP1 could play a significant role in SARS-CoV-2 infection as a facilitator of virus entry [13,18]. Expression data from COVID-19 patients revealed high expression of NRP1 [38] Cantuti-Castelvetri L et al, 2020 reported a maximum level of SARS-CoV-2 infection when NRP1, TMPRSS2, and ACE2 are expressed together [6]. All these recent studies signify the role of NRP1 as a therapeutic target for COVID-19 disease. NRP1 showed direct interaction with the spike protein of SARS-CoV-2 while proximal associations with its other proteins such as NSP4, NSP6, ORF3A, ORF7B, and Membrane protein (Fig 1A–1C). This data suggests that the binding of Spike protein with the NRP1 receptor could potentiate the infectivity of SARS-CoV-2 and can be inhibited by blocking the NRP1.

### Virtual screening

Datasets of _∼_ 10,000 compounds were screened against the b1 domain of the NRP1 receptor protein to identify the potential NRP1 inhibitors (S1–S4 Tables). We gathered the data about the CendR binding pocket of NRP1 which is critical for binding with the polybasic C-terminal sequence (of TQTNSPRRAR_OH_) of the S1 subunit of spike protein. The critical residues of NRP1 interacting with CendR motif are Tyr297, Trp301, Thr316, Ser346, Thr349, Lys351 and Tyr353. We screened our compound libraries against the NRP1 resulting in 366 hits. After virtual screening high energy scoring hits were evaluated for favorable interactions with critical residues of the NRP1 CendR binding site. Through these 20 compounds were shortlisted with binding energies ranging from -9.1 kcal/mol to -6.4 kcal/mol (S5 Table). These 20 compounds were further filtered to remove isomer and analogous resulting in 10 shortlisted compounds as shown in Table 1.

**Table 1:** Top 10 compounds binding energies and binding interactions.

S.No	Proteins	Binding Energies	Residues	Interactions
1	AZD3839	-9.1 kcal/mol	THR:316, SER:346, GLU:348, THR:349, LYS:351, TYR:353 TRP:301, ASP:320 TYR:297, SER:298	Van der Waals interactions Pi interactions Conventional Hydrogen Bonding
2	LY2090314	-8.0 kcal/mol	THR:299, ASN:300, GLU:348, THR:349 TYR:297, TYR:353, TRP:301 SER:298, SER:346, THR:316	Van der Waals interactions Pi interactions Conventional Hydrogen Bonding
3	CX-6258_HCL	-8.0 kcal/mol	SER:298, ASN:313, THR:316, SER:346, GLU:348, THR:349, LYS:351 TYR:297 TRP:301, TYR:353	Van der Waals interactions Pi interactions Conventional Hydrogen Bonding
4	Cryptotanshinone	-7.7 kcal/mol	THR:316, ASP:320, GLU:348, THR:349, LYS:351, GLY:414 TYR:297, TYR:353 TRP:301	Van der Waals interactions Pi interactions Conventional Hydrogen Bonding
5	Imperialine	-7.2 kcal/mol	THR:316, ASP:320, SER:346, THR:349, LYS:351, TYR:353, THR:413 TYR:297 TRP:301, GLU:348	Van der Waals interactions Pi interactions Conventional Hydrogen Bonding
6	Veratramine	-7.1 kcal/mol	THR:316, PRO:317, SER:346, GLU:348, THR:349, GLY:414, ILE:415, SER:416 TYR:297, TRP:301, TYR:353 ASP:320	Van der Waals interactions Pi interactions Conventional Hydrogen Bonding
7	PF_573228	-6.6 kcal/mol	SER:298, ASN:300, THR:316, SER:346, GLU:348, THR:349, LYS:351, GLY:414 TYR:297, TRP:301, TYR:353	Van der Waals interactions Pi interactions
8	Tanshinone IIA	-6.9 kcal/mol	THR:316, ASP:320, THR:349, LYS:351, GLY:414 TYR:297 TRP:301, TYR:353	Van der Waals interactions Pi interactions Conventional Hydrogen Bonding
9	Peiminine	-6.8 kcal/mol	ASN:300, THR:316, ASP:320, SER:346, GLU:348, GLY:414 TYR:297, TRP:301, and TYR:353	Van der Waals interactions Pi interactions
10	Hupehenine	-6.4 kcal/mol	THR:316, GLU:319, ASP:320, SER:321, SER:346 TYR:297, TRP:301, TYR:353	Van der Waals interactions Pi interactions

### Evaluation of ADMET properties

**Fig 2:**
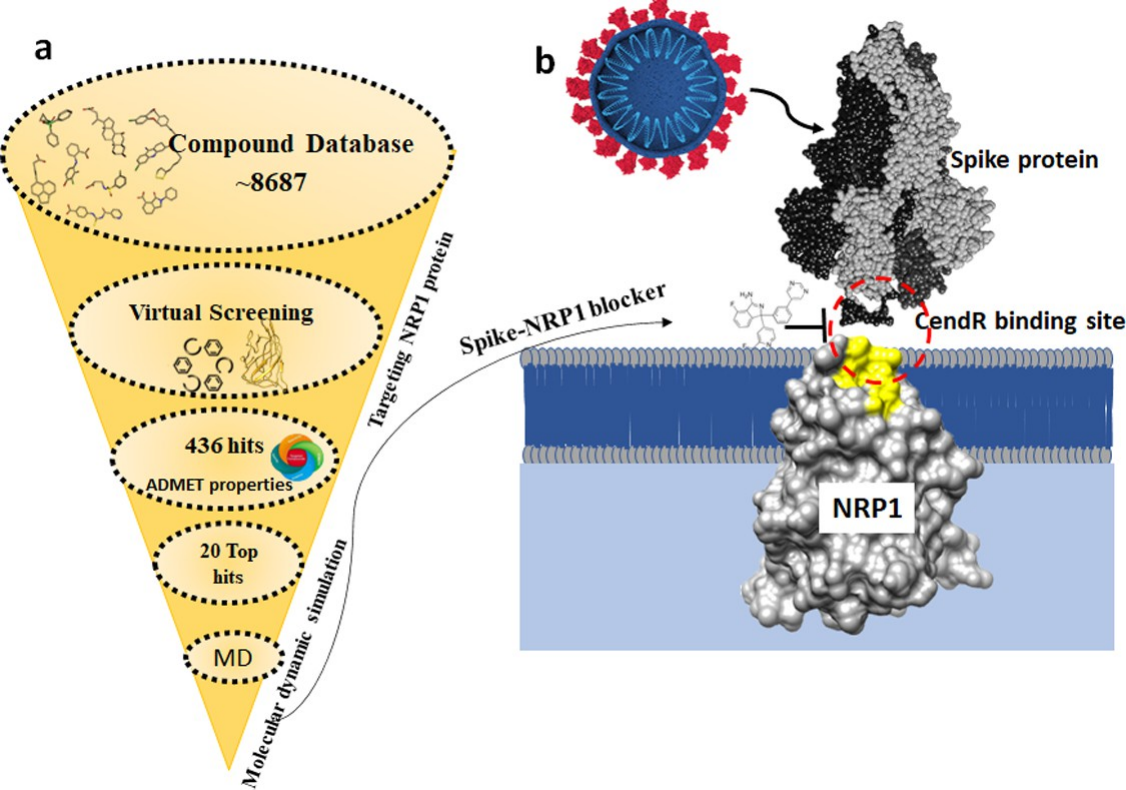
Schematic workflow of library screening. Insilico model of targeting S1 and NRP1 interaction.

The selected 10 compounds were evaluated for ADMET properties using the SwissADME tool and Molinspiration software Table 2. All the 10 compounds were predicted to have high gastrointestinal absorption, while 7 compounds resulted in a high b blood-brain barrier (BBB). In addition, all the compounds followed the Lipinski rule of five. The results of E-Dragon software showed 2 compounds with an ideal logS value of <-4. However, only AZD3839 and LY2090314 showed an ideal ADMET profile, fulfilling most of the drug-like properties. Thus, these compounds have the potential to act as a drug to inhibit the attachment of NRP1 and SARS-CoV-2 (Fig 2A and 2B).

**Table 2:** ADMET properties analysis of selected drug candidates.

Name	IUPAC Name	Molecular weight	Oral Bioavailability	Lipinski ROF	Solubility	BBB	GI Absorption	Log S	Log P	Log Kp	TPSA
AZD3839	(1S)-1-[2-(difluoromethyl)pyridin-4-yl]-4-fluoro-1-[3-(pyrimidin-5-yl)phenyl]-1H-isoindol-3-amine	439.48 g/mol	0.55	Yes	3.84e-02 mg/ml; 8.73e-05 mol/l	Yes	High	(-4.52)	-1.48	-7.31 cm/s	74.14 Å²
LY2090314	3-[9-fluoro-2-(piperidin-1-ylcarbonyl)-1,2,3,4-tetrahydro[1,4]diazepino[6,7,1-hi]indol-7-yl]-4-(imidazo[1,2-a]pyridin-3-yl)-1H-pyrrole-2,5-dione	514.55 g/mol	0.55	Yes	2.55e-02 mg/ml; 4.95e-05 mol/l	No	High	-3.22	-2.54	-8.01 cm/s	95.37 Å²
CX-6258_HCL	(3E)-5-Chloro-3-[(5-{3-[(4-methyl-1,4-diazepan-1-yl)carbonyl]phenyl}-2-furyl)methylene]-1,3-dihydro-2H-indol-2-one hydrochloride (1:1)	457.91 g/mol	0.55	Yes	2.18e-03 mg/ml; 4.76e-06 mol/l	Yes	High	(-4.36)	-4.36	-6.25 cm/s	65.79 Å²
PF_573228	N-[5-[(2S,5R,6S)-5-amino-6-fluorooxepan-2-yl]-1-methylpyrazol-4-yl]-2-(5,7-difluoro-2,3-dihydro-1-benzofuran-6-yl)-1,3-thiazole-4-carboxamide	493.50 g/mol	0.55	Yes	3.62e-02 mg/ml; 7.34e-05 mol/l	No	High	(-4.16)	-1.82	-7.79 cm/s	123.50 Å²
Imperialine	(3β,5α,17β)-3,20-Dihydroxycevan-6-one	429.64 g/mol	0.55	Yes	5.06e-03 mg/ml; 1.18e-05 mol/l	Yes	High	(-4.16)	-3.32	-6.19 cm/s	60.77 Å²
Hupehenine	(3β,5α,6β,17β)-Cevane-3,6-diol	415.65 g/mol	0.55	Yes	5.15e-04 mg/ml; 1.24e-06 mol/l	Yes	High	(-5.09)	(3.64)	-4.90 cm/s	43.70 Å²
Veratramine	(3β,23R)-14,15,16,17-Tetradehydroveratraman-3,23-diol	409.60 g/mol	0.55	Yes	3.04e-03 mg/ml; 7.42e-06 mol/l	Yes	High	(-5.71)	-4.57	-5.72 cm/s	52.49 Å²
Tanshinone IIA	1,6,6-Trimethyl-6,7,8,9-tetrahydrophenanthro[1,2-b]furan-10,11-dione	288.30 g/mol	0.55	Yes	3.23e-02 mg/ml; 1.12e-04 mol/l	Yes	High	(-4.26))	-2.92	-5.86 cm/s	47.28 Å²
Cryptotanshinone	(1R)-1,6,6-Trimethyl-1,2,6,7,8,9-hexahydrophenanthro[1,2-b]furan-10,11-dione	296.36 g/mol	0.85	Yes	1.58e-02 mg/ml; 5.33e-05 mol/l	Yes	High	(-4.48)	-4.32	-5.41 cm/s	43.37 Å²
Peiminine	(3β,5α)-3,20-Dihydroxycevan-6-one	429.64 g/mol	0.55	Yes	5.06e-03 mg/ml; 1.18e-05 mol/l	Yes	High	(-4.16)	-3.32	-6.19 cm/s	60.77 Å²
AZD3839	(1S)-1-[2-(difluoromethyl)pyridin-4-yl]-4-fluoro-1-[3-(pyrimidin-5-yl)phenyl]-1H-isoindol-3-amine	439.48 g/mol	0.55	Yes	3.84e-02 mg/ml; 8.73e-05 mol/l	Yes	High	(-4.52)	-1.48	-7.31 cm/s	74.14 Å²

### Interaction analysis of shortlisted compounds with NRP1

AZD3839 is a human BACE1 inhibitor and it is brain-permeable. Jeppsson *et al* 2012 reported that AZD3839 reduced the level of amyloid beta in the brain and it has the potential to treat Alzheimer’s and other brain-related disorders [39].

**Fig 3:**
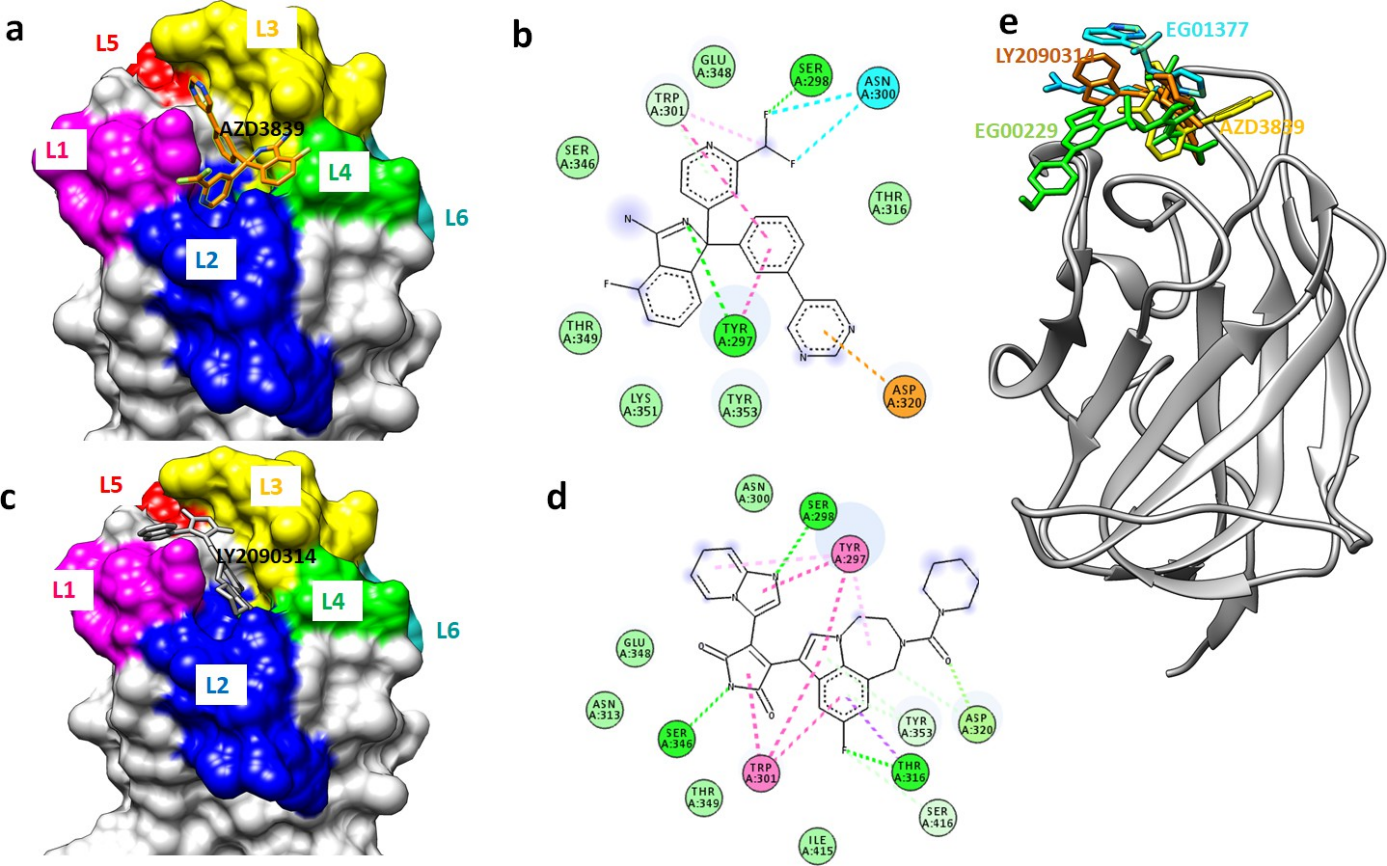
Binding mode analysis of shortlisted drugs with NRP1. **(a)** Surface representation of b1 domain of NRP1 with six juxta posed loop region demarcating the binding groove. AZD3839 is shown in orange stick model **(b)** 2D representation of AZD3839-NRP1 interactions. AZD3839 is shown in the grey stick model while the binding residues are in the disc **(c)** surface model and **(d)** 2D representation of LY2090314-NRP1 interaction **(e)** Comparative analysis of known inhibitors of NRP1 with AZD3839 and LY2090314.

In the present study virtual screening results showed high affinity binding of AZD3839 with NRP1 receptor with binding free energy of -9.1 kcal/mol. The L1 loop at the opening of the binding groove was tilted deep into the groove to make interactions with the AZD3839. The pyrimidine moiety of AZD3839 formed conventional hydrogen bonds with the hydroxyl group of the polar side chain of Tyr297 with a hydrogen bond distance of 1.37Å. Similarly, the OG group of Ser 298 formed a hydrogen bond with fluorine moiety of AZD3839 with a hydrogen bond distance of 1.36Å. The OD and HD group of Asn300 formed two halogen bonds with the difluoromethane moiety of AZD3839. The side chain of Trp301 lies deep into the binding groove and forms pi-pi-staking interactions. The residues of the L2, L3, and L4 loops (Ser346, Glu348, Thr349, Lys351, and Tyr353) demarcate hydrophobic grooves and form Vander walls interaction with the AZD3839. This overall binding affinity is driven by these hydrophobic interactions (Fig3A and 3B).

LY2090314 is a potent and selective ATP-competitive inhibitor of glycogen synthase kinase-3 and is currently in clinical trials for cancer therapy [40]. Luca Murer et al reported LY2090314 as a remarkable inhibitor in their hCoV-229E-GFP screen with an EC_50_ of 2 nM. In addition, it helps in decreasing the human MYCN amplified and non-amplified neuroblastoma cell lines development *in vitro*. Most of the anticancer drugs are in clinical trials for COVID-19 [41]and repurposed as antiviral drugs against COVID-19 infection [42]. Detailed interaction analysis of LY2090314 with NRP1 CendR binding site revealed high binding affinity with binding free energy of -8.0kcal/mol. L1 (Ser298), L3 (Ser346), and L5 (Thr316) loop residues formed conventional hydrogen bonds with the LY2090314. Corban beta of serine 298 and serine 346 form hydrogen bonds (1.4Å and 1.3 Å) with an amide group of inhibitors. While the Thr316 formed a hydrogen bond with the fluorine group. In addition, Tyr353 forms a h carbon-hydrogen bond. The hydrophobic groove (Asn300, Ans313, Thr349, and Glu 348) involving five loops (L1-L5) formed Vander Waals interactions with LY2090314 to keep pretty well into the CendR binding site (Fig 3C and 3D).

To further validate the affinity and residual interaction of our shortlisted hits, known NRP1 inhibitors EG00229 [36] and EG01377 [37] were selected to dock against the b1 domain of NRP1. These inhibitors were reported to inhibit the binding of NRP1 with VEGF [43]. This docking was performed to gain confidence in the binding behavior of our identified hits and our docking procedure. The known inhibitors and our identified hit docked well with equal affinity in the CendR binding motif of the NRP1 protein (Fig 3E).

### Molecular dynamic simulation

Molecular dynamic simulations were performed to measure the stability and structural behavior of the NRP1 in unbound and bound states with AZD3839 and LY2090314. Molecular dynamic simulations were run for 100ns using GROMACS package version 5.0.5 [29]. To measure the fluctuation and stability of the NRP1 protein in the apo state and bound with shortlisted hits, root mean square fluctuation (RMSF) and root mean square deviation (RMSD) were calculated (S6 Table). Analysis of the results indicated overall convergence of the energies and stabilized systems.

**Fig 4:**
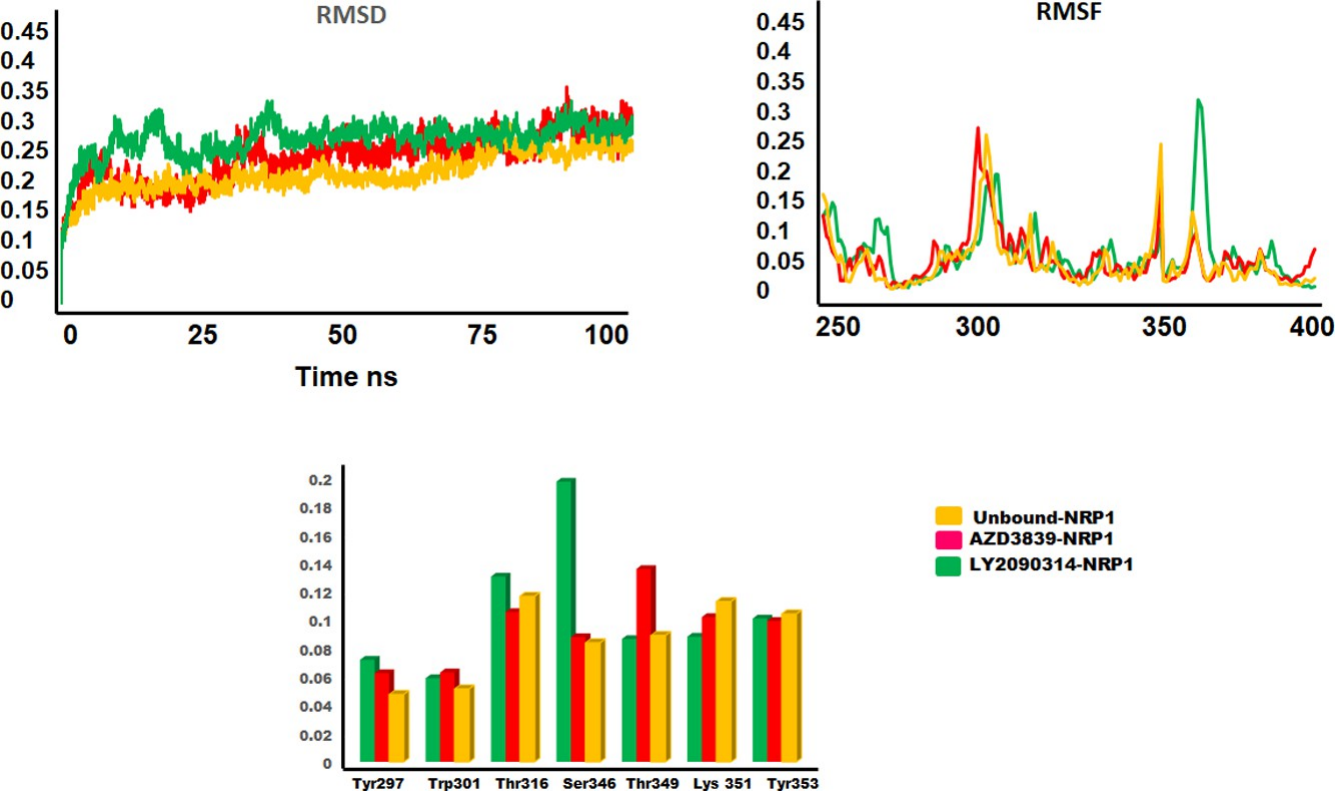
Molecular dynamic simulation of AZD3839 and LY2090314 bound to NRP1 compared to unbound NRP1. (**a**) root mean square deviation (**b**) root mean square fluctuation and (**c**) binding residue analysis.

RMSD was calculated at 100 ns for AZD3839-NRP1 and LY2090314-NRP1 using the unbound NRP1 protein as a reference. The AZD3839-NRP1 system started from approximately 0 nm and stabilized between approximately 0.15–0.25 nm until 30 ns, with a slight increase in stability to approximately 0.28 nm. The system then stabilized between approximately 0.2–0.3 nm from 40 ns until 100 ns. In the LY2090314-NRP1 complex, an increase in RMSD value was observed from approximately 0.15 nm to 0.3 nm until 45 ns, and for the rest of the simulation, the system stabilized between approximately 0.2–0.3 nm. The measured average RMSD between the bound and unbound NRP1 was below 2 Å, suggesting the stability of the system (Fig 4A).

Next, the RMSF analysis of AZD3839-NRP1 and LY2090314-NRP1 complexes, compared to unbound NRP1, indicated increased fluctuations in the first 50 residues. The loop region near the binding site exhibited noticeable fluctuations, whereas the CendR binding motif residues (Tyr297, Trp301, Lys351, Tyr353, Ser346, Thr349) remained stable, showing only slight changes in orientation to aid in binding (Fig 4B and 4C).

**Fig 5:**
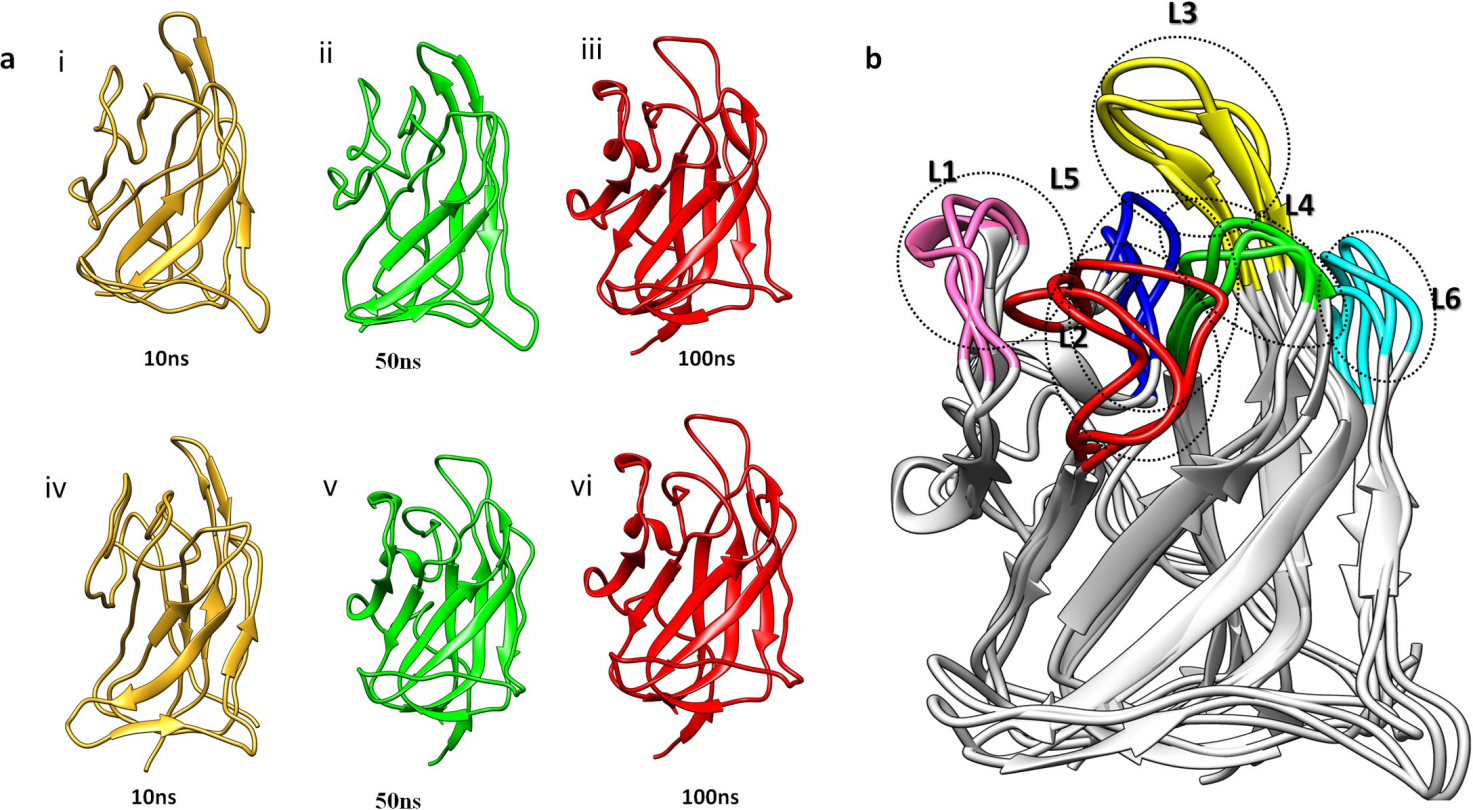
Conformational changes in b1 domain of NRP1 upon binding with AZD3839 and LY2090314. **(a)** i, ii and iii NRP1-AZD3839 and iv, v, vi NRP1-LY2090314 at 10ns, 50ns and 100ns. (**b**) comparative analysis of conformational changes of unbound NRP1 with AZD3839 and LY2090314 bound NRP1.

Dynamic trajectories of AZD3839-NRP1 and LY2090314-NRP1 were generated at different time scales to measure the important structural and conformational twists in the bound state of NRP1 in comparison to the unbound state. The L1 and L3 showed significant outward movement to open the binding groove with conformational space assessed by AZD3839. While the L2, L3, and L5 bend inwards to widen the cavity and the resulting change in orientation of the binding site residues to attain a pose aid in binding to AZD3839. Similarly, the overlap of unbound and bound (LY-2090314) revealed conformational switches in the L1-L5 loops critical for holding the ligand inside the binding groove. Significant conformational poses were adopted by binding residues to make several numbers of interactions with the LY-2090314 ligand (Fig 5A and 5B).

### MM-PBSA calculations

To estimate the interaction energies of protein-ligand complexes binding free energy MM-PBSA was calculated. The MD combined with MM-PBSA integrates conformational fluctuations and entropies to the binding energy. The binding of ligands with proteins is associated with various free energies. Initially, we calculated the ΔE_MM_, ΔG_polar_, and ΔG_nonpolar_ energies separately for the last 10ns simulation trajectories of each system and then combined them to predict the binding free energy of individual systems. MM-PBSA energy calculation of AZD3839 in complex with NRP1 revealed a relatively higher binding free energy of -297.36 kcal/mol as compared to the LY-2090314-NRP1 system that showed binding energy of -211.47 kcal/mol (Table 3).

**Table 3:** The MM-PBSA energies of NRP1-AZD3839 and NRP1-LY2090314 complexes.

Complexes	MM-PBSA (kcal/mol)	ΔG Bind vdWc (kcal/mol)	ΔG Solv GBd (kcal/mol)	Electrostatic Energy (kcal/mol)
**NRP1-AZD3839**	-15.04	-25.362	11.232	-12.647
**NRP1-LY2090314**	-14.35	-23.843	10.722	-11.103

### Comparative docking of apo and NRP1-drug complexes with spike protein

**Fig 6:**
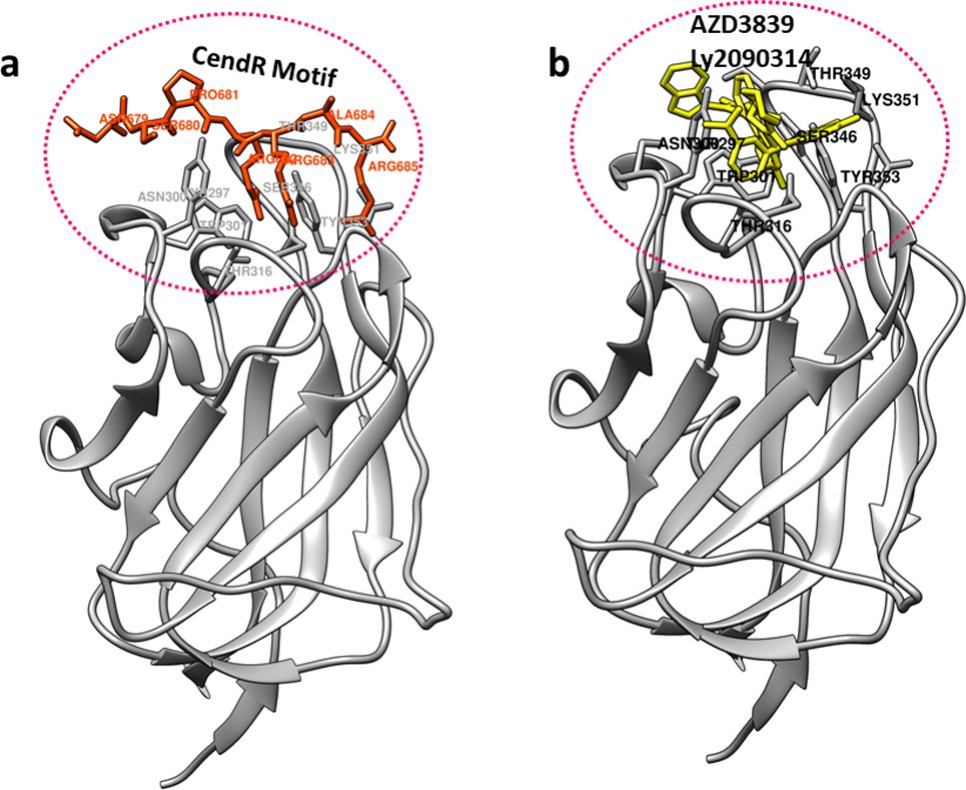
Comparative binding of CendR motif and shortlisted compounds of the present study. (**a**) Binding analysis of CendR motif at the binding site of NRP1. CendR motif is shown in the orange stick model and the binding residues of NRP1 is shown in the grey stick model. (**b**) Binding of shortlisted compounds on the same binding site.

Daly et al. reported that CendR motif of S1 directly binds to NRP1, and blocking this interaction reduced the entry and infectivity of SARS-CoV-2. Through molecular docking analysis, a hypothetical model was proposed that blocking the NRP1 CendR binding site will diminish or result in the loss of interaction between NRP1 with S1 of the spike protein. Initial docking of NRP1 with the S1 (CendR binding motif) revealed binding with the CendR binding site of NRP1 (Fig 6A). In contrast, docking analysis of inhibitor-bound NRP1 with the CendR motif of S1 showed a loss of interaction between the NRP1 receptor and a spike protein as both compete for the same binding site and thus could reduce the NRP1-assisted virus infectivity (Fig 6B).

## Discussion

COVID-19 was declared a public health emergency by WHO. The continuous spread of SARS-CoV-2 has resulted in serious global concerns. During this pandemic situation, computational high-throughput screening of small compound libraries and drug repurposing has accelerated the process of drug discovery against SARS-CoV-2 at the entry, replication, and maturation, stages. Viral entry is considered as a promising therapeutic intervention, as it is the first step in its pathogenesis. The main focus is on inhibiting virus-receptor interaction and its fusion with the host cell membrane, which enhances viral genome delivery.

The SARS-CoV-2 spike protein comprises two components: S1, known as the receptor-binding subunit, and S2, the fusion subunit. Through the S1 subunit, the spike protein attaches to the b1 domain of NRP1, while the S2 subunit facilitates viral fusion with the target cell membrane. NRP1 catalyzes proteolytic cleavage, aiding viral fusion with the cellular membrane and entry into target cells [44] so NRP1 could be a strong candidate against COVID-19. Therefore, in the present study, docking was performed against the amino acids in the b1 region of NRP1 (Tyr297, Trp301, Thr316, Ser346, Thr349, Lys351, and Tyr353), which are important for its interaction with spike protein [18]. The main focus was on preventing this interaction because it has been shown that the infectious process can be reduced by blocking the interaction between NRP1 and S1 (spike) protein [16,18,45].

In this study, we initially screened a library of ∼10,000 compounds against the b1 domain of NRP1 (Thr316, Asp320, Ser346, Thr349, and Tyr353) CendR binding motif important for binding with S1 subunit of spike protein Top ten scoring compounds (Table 1) showed interaction with Tyr297, Asn300, Trp301, Thr316, Gly318, Glu319, Asp320, Ser321, Arg323, Glu348, Lys351, Tyr353, Trp411, Thr41 residues of NRP1. These results suggest that this list of ligands could be considered as good inhibitors for NRP1. Further filtering based on SAR properties AZD3839, and LY2090314 were found most suitable inhibitors of NRP1 (Table 2). AZD3839 is placed well within the deep grove formed by the L1-L5 loops. Tyr297 and Ser298 of L1 loop suspended into the groove and made a conventional hydrogen bond. While the other side of the groove encompassing L2, L3, and L4 makes strong Vander Waals interactions. Most of the L1 and L3 residues involved in binding with AZD3839 form a basic cap of the binding groove and also these residues are involved in cell adhesion activity of the NRP1.

AZD3839, a synthetic compound, is reported to be used in the treatment of Alzheimer’s disease as it inhibits the activity of β-site amyloid precursor protein cleaving enzyme1 (BACE1), which is one of the key enzymes involved in the processing of amyloid precursor protein (APP) and the formation of amyloid β peptide (AβP) species [39]. The binding pocket of NRP1 and BACE1 was compared to identify the structural similarity that justifies the interaction of AZD3839 with NRP1. Comparative analysis of the binding pocket showed that NRP1 and BACE1 share 4 common residues and suggested that AZD3839 could be a good inhibitor of NRP1. Moreover, as AZD3839 can cross the blood-brain barrier so it can block the NRP1 in sensory olfactory neurons and block the entry of the virus into the brain.

The other shortlisted compound LY2090314 docked well within the interloop cleft formed by L1-L5. L1 and L3 loops at the opening of the cavity were extended towards the interloop cleft and residues of this hydrophobic cap made several interactions with LY20290314 with high binding affinity. LY2090314 is a potent inhibitor of glycogen synthase kinase-3 (GSK-3), an oncogene, and is highly expressed in neuroblastoma [46,47]. Murer L etal in 2021 reported the remarkable effect of LY2090314 as a potent compound against human coronaviruses [48] which further strengthens our screening results. Comparative analysis of NRP1 and GSK3 binding pocket showed that both proteins share Try, Thr, and Lys residues in their pocket, hence the identified inhibitor in our screen is significant to block the CendR biding site of NRP1 and thus could reduce the SARS-CoV-2 infection. Extensive molecular dynamics studies further elaborated the important structural behavior of important binding site residues and loop regions decorating the hydrophobic binding groove of NRP1. Among the six juxtaposed loop regions (L1-L6) the most prominent L1 and L3 loops were drawn towards the binding cleft and played a remarkable role in stabilizing the interaction between NRP1 and shortlisted candidate drug molecules (Fig 6B). The binding site residues of NRP1 CendR binding site (Tyr297, Ser298, Asn300, Trp301, Asp320, Ser346, Glu348, Thr349, Lys351, Tyr353) showed consistent behavior throughout the dynamic simulations hence proving that these shortlisted inhibitors could significantly block the NRP1 receptor. RMSD and RMSF behavior showed consistency throughout the simulation trajectories.

Further, a hypothetical model was proposed that depicts the loss of binding between NRP1 and CendR binding motif of S1 (spike). Docking of solo and inhibitor-bound NRP1 with the CendR motif of S1 showed that the CendR motif of S1 and inhibitor share the same binding site on the b1 domain of NRP1 and inhibitors identified in the present study could block the CendR binding motif and diminish the binding of S1 and thus could be an attractive strategy to stop viral infection. As these two drugs (AZD3839, LY20290314) are already in clinical trials and cross the blood-brain barrier these would be good candidate drugs to stop viral infection of the brain. Most of the studies reported that viruses can get into the brain through the NRP1 receptor [15]. The opportunity to develop more effective and selective drugs against NRP1 is still open and it can potentially reduce the SARS-CoV-2 infection [15,16,49].

The COVID-19 pandemic has posed a major threat to public health due to the absence of effective drugs against SARS-CoV-2. Despite the emergency approval by the WHO, questions remain about the efficacy of vaccines, as cases of reinfection continue to occur [50]. This research highlights a critical advancement in our understanding of the mechanisms underlying SARS-CoV-2 infectivity and presents a novel therapeutic strategy. The extensive screening of a small molecule compounds library and subsequent molecular dynamics simulations identified two promising compounds, AZD3839 and LY2090314, which exhibited strong binding affinity and stability with the NRP1 b1 domain. The use of molecular dynamics simulations and MM/GBSA calculations provides a high level of predictive accuracy for binding stability and interaction efficacy, which are crucial for the validation of potential inhibitors. This approach broadens the scope of potential anti-SARS-CoV-2 interventions and underscores the importance of targeting host-virus interactions to mitigate viral infectivity, paving the way for new antiviral drug development. The limitation of the study is the experimental validation of the screening results due to a lack of experimental support. However, the identified results are significant and could support further experimental validation.

## Conclusion

The study found that targeting the interaction between the SARS-CoV-2 spike protein and the neuropilin-1 (NRP1) b1 domain could effectively reduce viral infection. By screening a library of about 10,000 compounds, AZD3839 and LY2090314 were identified as promising candidates. These compounds exhibited strong and stable binding to the b1 domain of NRP1, as shown by molecular dynamics simulations and MM/GBSA calculations. Computational modeling indicated that both compounds could hinder the interaction between NRP1 and the spike protein, potentially decreasing SARS-CoV-2 infectivity. Therefore, AZD3839 and LY2090314 may serve as potential drug candidates for COVID-19 treatment.

## Supporting information

S1 Table Inhibitor library. (CSV)

S2 Table Natural organic compound library. (CSV)

S3 Table Natural product library. (CSV)

S4 Table OTAVA library. (CSV)

S5 Table ADMET Top20 compounds. (XLSX)

S6 Table RMSD and RMSF. (XLSX)

S1 Graphical abstract (TIF)
