# Correction: *Insilico* targeting of virus entry facilitator NRP1 to block SARS-CoV2 entry

**DOI:** 10.1371/journal.pone.0330614

**Published:** 2025-08-20

**Authors:** Nousheen Bibi, Maleeha Shah, Shahzad Khan, Muhammad Shahzad Chohan, Mohammad Amjad Kamal

The Funding statement for this article is incorrect. The correct Funding statement is as follows: The author would like to thank the Deputyship for Research and Innovation at King Faisal University, Saudi Arabia (grant number GRANT KFU242004).

## References

[1] Bibi N, Shah M, Khan S, Chohan MS, Kamal MA. *Insilico* targeting of virus entry facilitator NRP1 to block SARS-CoV2 entry. PLoS One. 2025;20(2):e0310855. doi: 10.1371/journal.pone.0310855 39908250 PMC11798527

