# Supplementary figures and images for: Insilico targeting of virus entry facilitator NRP1 to block SARS-CoV2 entry
Source: PLoS One. 2025 Feb 5;20(2):e0310855. doi: 10.1371/journal.pone.0310855 (PMC11798527; doi:10.1371/journal.pone.0310855)

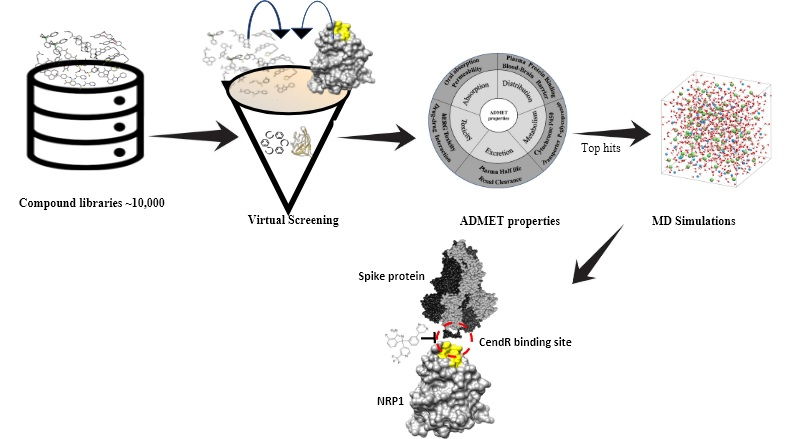

Supplement: S1 Graphical abstract — (TIF) [file pone.0310855.s007.tif]
